# The Role of Iron Chelation Therapy in Colorectal Cancer: A Systematic Review on Its Mechanisms and Therapeutic Potential

**DOI:** 10.1002/cam4.71019

**Published:** 2025-07-03

**Authors:** Gihani Vidanapathirana, Md Sajedul Islam, Sujani Gamage, Alfred K. Lam, Vinod Gopalan

**Affiliations:** ^1^ School of Medicine and Dentistry Griffith University, Gold Coast Campus Southport Queensland Australia; ^2^ Department of Medical Laboratory Science, Faculty of Allied Health Sciences University of Peradeniya Peradeniya Sri Lanka; ^3^ Department of Biochemistry and Biotechnology, Faculty of Biological Sciences University of Barishal Barishal Bangladesh; ^4^ Faculty of Health Sciences and Medicine Bond University Gold Coast Queensland Australia; ^5^ Pathology Queensland Gold Coast University Hospital Southport Queensland Australia

**Keywords:** anticancer effects, Colorectal cancer, iron chelation, systematic review

## Abstract

**Background:**

Despite significant therapeutic advancements in recent decades, colorectal cancer (CRC) continues to exhibit high rates of mortality and morbidity. Chemoresistance and cancer recurrence remain substantial challenges, underscoring the need for novel treatment approaches. Iron chelation therapy has gained profound interest over the years as a potential cancer treatment, leveraging the increased iron demand by tumors. This review evaluates the effects of iron chelation therapy on CRC progression and the underlying mechanisms.

**Method:**

A comprehensive review of in vivo and in vitro studies was conducted to assess the effectiveness of iron chelation therapy in CRC. The literature search covered PubMed, Scopus, Medline (via Web of Science), and EMBASE between January 1995 and March 2024.

**Results:**

Several in vitro and in vivo studies have investigated the impact of iron chelators, such as deferoxamine, deferasirox, thiosemicarbazone‐based chelators, quilamine‐based chelators, and other novel compounds on CRC. Natural plant extracts with iron‐chelating properties have also been explored as potential treatments. Most studies indicate that iron chelation can inhibit the proliferation of colon cancer cells, though some studies suggest cancer‐promoting effects. Mechanistically, iron chelation affects several hallmarks of CRC by modulating histone methylation, upregulating NDRG1, and influencing the Wnt/β‐catenin and p53 signaling pathways. However, certain iron chelators may inhibit TRAIL‐mediated apoptosis and activate the hypoxia‐inducible factor (HIF), potentially accelerating CRC progression.

**Conclusion:**

Future exploration of iron chelation therapy in CRC should focus on extensive in vitro, in vivo, and clinical studies to elucidate the precise mechanisms involved. A deeper understanding of the genetic and cellular alterations induced by iron chelation will enhance the development of effective therapeutic strategies for CRC.

## Graphical abstract

Iron chelation is a promising yet underexplored strategy for colorectal cancer (CRC) treatment. Iron chelators have a dual role in inhibiting and potentially promoting cancer progression. Iron chelation affects key CRC pathways, including histone methylation, NDRG1 upregulation, Wnt/β‐catenin, and p53 modulation. Comprehensive in vitro and in vivo analyses reveal the need for additional studies to fully understand the antiproliferative mechanisms and cellular targets of iron chelators.

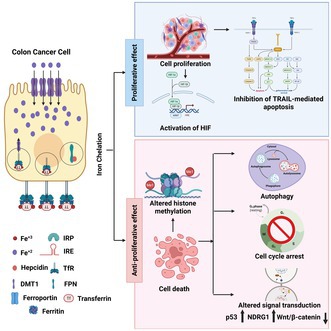

## 1 Introduction

Colorectal cancer (CRC) continues to exhibit high mortality and morbidity rates despite advancements in therapeutic approaches. According to World Health Organisation (WHO) statistics, CRC is the third most prevalent cancer worldwide and the second leading cause of cancer‐related deaths [1]. It is predicted that by 2040, the burden of CRC will escalate to 3.2 million new cases and 1.6 million deaths per year [1].

Treatment strategies for CRC vary depending on the stage of the disease. Surgical excision, particularly minimally invasive procedures, remains the primary treatment option for most cases [2]. This may be supplemented with adjuvant therapies, such as targeted therapy and immunotherapy, for patients with stage II and III CRC [3]. However, the clinical application of these adjuvant therapies is often limited [3]. The prognosis of CRC is closely linked to the stage at diagnosis, with surgical resection identified as the primary curative treatment, yielding better outcomes for approximately 80% of CRC patients with localized cancers [4]. Despite these primary and adjuvant treatments, recurrence occurs in nearly 20%–40% of patients, highlighting the necessity for postoperative surveillance [4, 5]. Moreover, the 5‐year relative survival rate is significantly higher in patients diagnosed with localized cancer stages compared to those with metastatic disease [6]. Patients who develop cancer recurrence face an increased risk of death, underscoring the importance of considering recurrence risk when selecting appropriate therapeutic approaches.

The American Society for Cancer Research has identified several modifiable and non‐modifiable risk factors associated with CRC. Modifiable risk factors include being overweight or obese, a diet high in red and processed meats, cooking meat at high temperatures, smoking, and excessive alcohol consumption. Non‐modifiable risk factors encompass age over 50 years, male sex, a history of colorectal polyps, and a history of inflammatory bowel disease [7]. Additionally, iron, although essential for survival, has been implicated in the pathogenesis and progression of CRC [8, 9, 10].

### 1.1 Iron in Cellular Metabolism

Iron is an essential element for normal cellular metabolism. It is found in the active centers of enzymes and oxygen‐carrying proteins such as hemoglobin. Iron is a critical component of cytochrome a, b, and c, cytochrome oxidase, and the iron–sulfur (Fe‐S) complexes involved in the oxidative phosphorylation cycle for ATP synthesis [8, 11]. During DNA synthesis, iron serves as a cofactor in ribonucleoside reductase, which catalyzes the formation of deoxyribonucleotides from ribonucleotides. Thus, it is crucial to maintain a constant supply of iron to support the functions of all the organs [11]. However, there is substantial evidence linking iron to various diseases, including cancers [8, 12].

Several iron regulatory proteins, responsible for iron import, export, and storage, tightly regulate the cellular iron pool. Transferrin (Tf), transferrin receptors (TfR), and divalent metal transporter I (DMT1) have a crucial role in the iron import to the cell, while ferritin acts as the iron storage protein in the cell. Ferroportin helps export iron from cells. Hepcidin, which is a liver‐derived hormone, regulates the systemic iron homeostasis [13]. In CRC, abnormal iron metabolism is well established, as these cells exhibit a higher demand for iron due to the rapid DNA synthesis during cell growth [8, 12]. This increased demand is met by the upregulation of iron regulatory proteins and transferrin receptors in CRC cells [14]. Consequently, CRC cells are sensitive to iron depletion within the tumor microenvironment, making iron chelation a promising approach to cancer therapy.

### 1.2 Iron Chelation Therapy

Iron chelation therapy, aimed at preventing iron overload, has expanded interest in recent years as a potential primary or adjuvant treatment option for various cancers [15]. This iron chelation is a targeted treatment in which the chemical agents bind and remove excess iron from the body. These chelating agents tightly bind the free iron inside the cells, forming water‐soluble complexes [16]. This bound iron is then excreted through urine or bile. When iron is bound to a chelating agent, its chemical reactivity is reduced and becomes more stable, facilitating the excretion. Numerous studies have demonstrated the antiproliferative effect of iron chelators against various tumor types [17, 18]. Conversely, some studies have shown that iron chelators promote the invasion and migration of some aggressive cancer cells [19, 20, 21]. These results reveal that iron chelators have varying effects on different cancer cell phenotypes, emphasizing the need for further investigation. However, the potential use of iron chelators in CRC has not been extensively studied.

Iron metabolism is altered in CRC to enhance proliferation or metastasis [9]. Hence, theoretically, iron deprivation via chelation represents a potential chemotherapeutic strategy. Nevertheless, both in vitro and in vivo studies have revealed that while iron chelation can suppress some malignancies, such as colorectal cancers, breast cancers, and lung cancers [10], conversely, some iron chelators can activate oncogenic signaling pathways that escalate tumor growth, a phenomenon particularly evident in aggressive breast cancer cells [19, 22]. The mechanisms underlying these contrasting effects are still not fully understood. This highlights the need for comprehensive studies to elucidate how iron depletion exerts anti‐proliferative effects on specific cancer types, which could aid in the development of targeted chemotherapy strategies for improved patient prognosis.

### 1.3 Iron Chelators

Iron chelation therapy was initially developed to treat certain hematological diseases such as thalassaemia, transfusion‐induced anemia, hemochromatosis, and hematological malignancies [23]. Iron chelators are natural or synthetic molecules that bind to iron with high affinity. Various synthetic and plant‐based compounds have been utilized for therapeutic purposes due to their iron‐chelating properties.

Iron chelators can be categorized into different classes based on their structure and functional capacity (Table 1). Currently, three iron chelators—deferoxamine, deferiprone, and deferasirox—are approved by the US Food and Drug Administration (FDA) for clinical use. In addition, several novel iron chelators have been synthesized and investigated for potential therapeutic applications. Several plant‐derived extracts have also been studied for their capacity for iron chelation, which is then linked to potential anticancer activities [30, 31, 32].

**TABLE 1:** Commonly used iron chelators in clinical practice and research.

Iron chelator	Chemical structure	Source	Mode of action	Ref.
Deferoxamine (DFO)	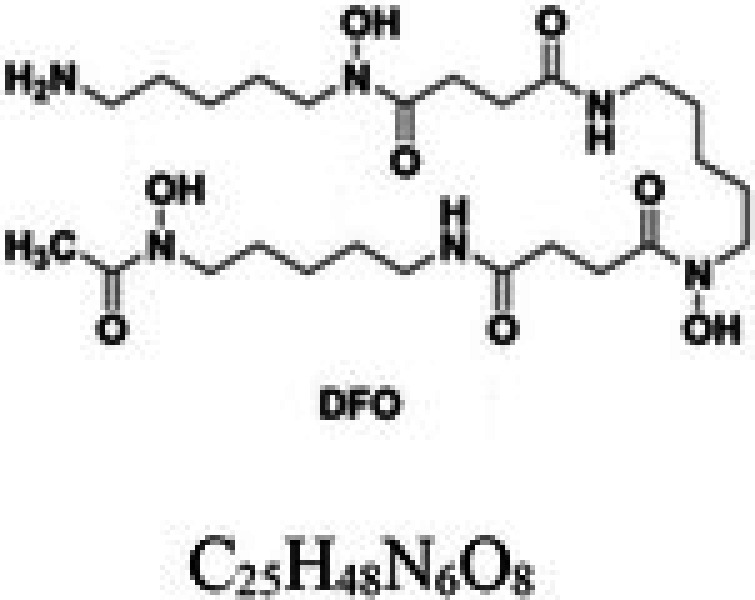	Siderophores, produced by microorganisms *Streptomyces pilosus*	Hexadentate ligand forming a 1:1 bond with Fe^3+^ High affinity for iron	[24]
Deferasirox (DFX)	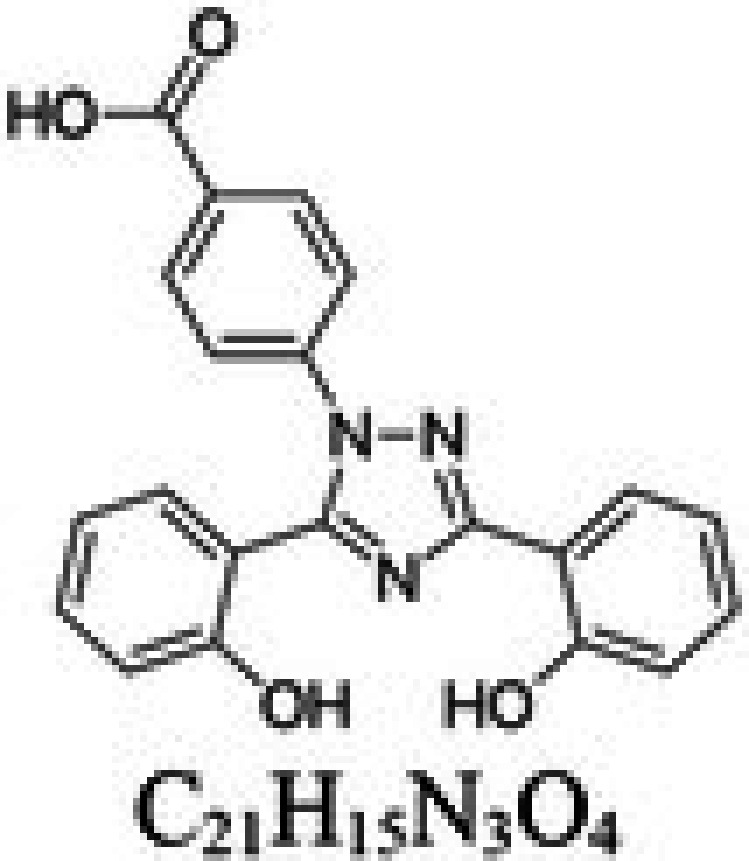	Synthetic iron chelator in the class of triazoles	Tridentate ligand, which forms a 2:1 bond with iron. DFX is capable of binding to either Fe^2+^ or Fe^3+^	[25]
Thiosemicarbazones				
3‐aminopyridine2‐carboxaldehyde thiosemicarbazone (3‐AP) or Triapine	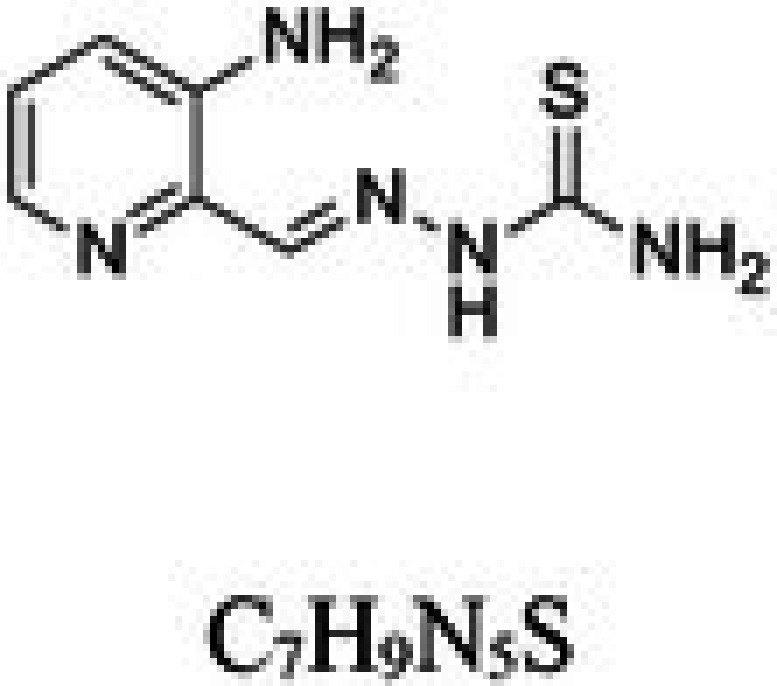	Synthetic heterocyclic carboxaldehyde thiosemicarbazone	Triapine inhibits the enzyme ribonucleotide reductase in which Triapine binds to and destabilizes the central Fe^2+^ in the R2 subunit, inactivating ribonucleotide reductase	[26]
Di‐2‐pyridylketone thiosemicarbazones (DpT)	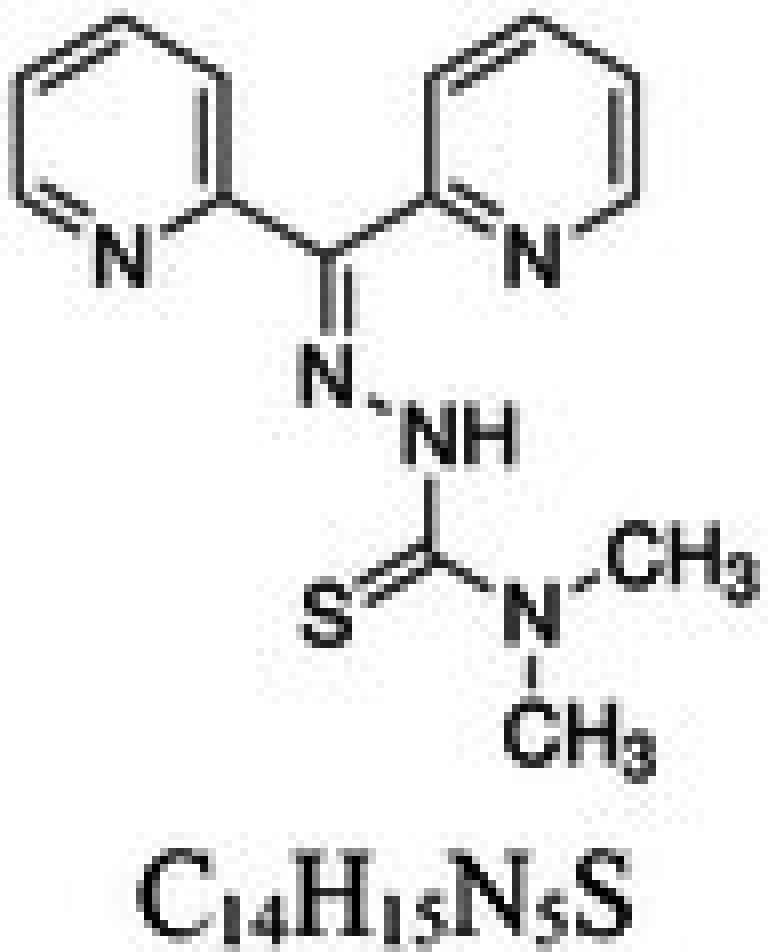	Newer synthetic thiosemicarbazones	Fe chelation and redox cycling of its Fe complex to generate reactive oxygen species	[27]
2‐benzoylpyridine thiosemicarbazones (BpT)	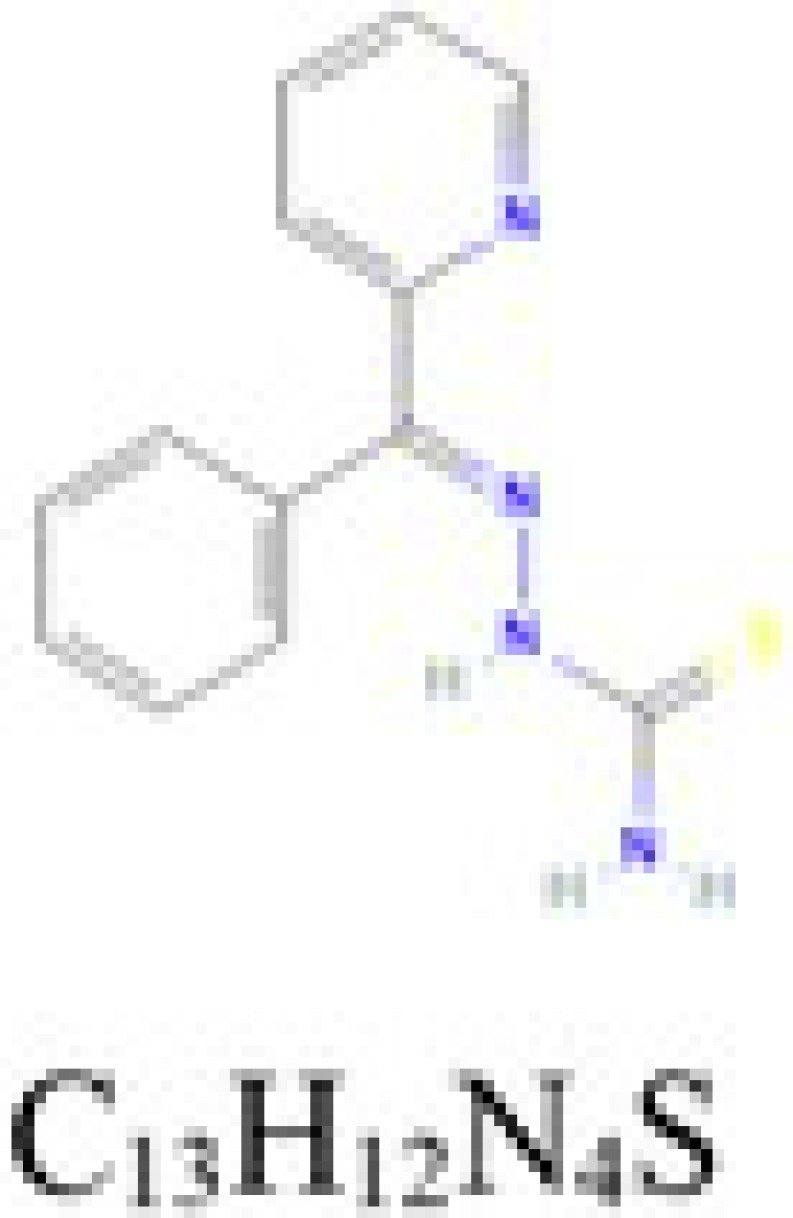	Newer synthetic thiosemicarbazones	Fe^2+^ and Fe^3+^ chelating redox cycling to generate reactive oxygen species	[28]
Pyridoxal isonicotinoyl hydrazone (PIH) analogues	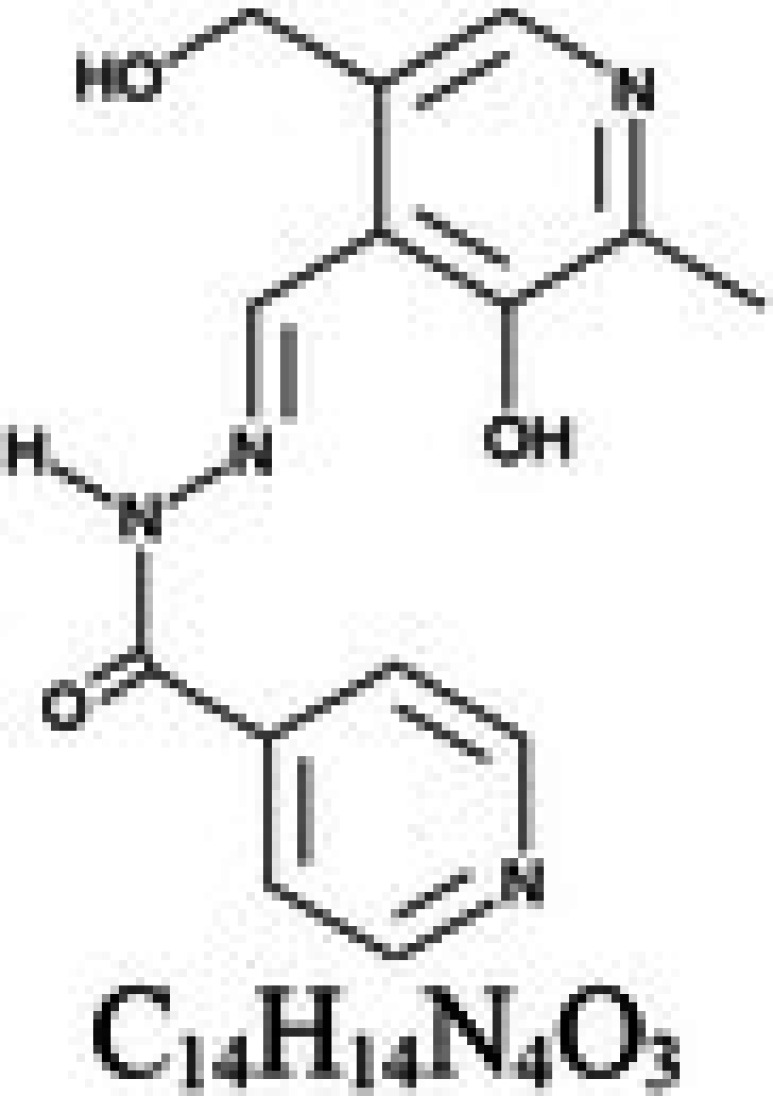	Class of tridentate iron chelators	Lipophilic nature and higher affinity for iron chelation and targeting the R2 subunit of ribonucleotide reductase	[29]

Although iron chelation has been extensively reviewed in cancer therapy, its specific impact on CRC remains underexplored. Hence, this review aims to systematically collate available literature regarding iron chelation therapy in CRC, with a specific focus on both synthetic and natural iron chelators and their anticancer effects. Moreover, the underlying mechanisms responsible for the anticancer effect of these different iron chelators are examined in detail.

## 2 Methodology

The initial research question was searched using the terms “Iron Chelation” and “CRC”, targeting relevant articles published between 1995 and 2024. A comprehensive search strategy was developed for MEDLINE using the Medical Subject Headings (MeSH) terms. The target articles were retrieved from four major databases: PubMed, Scopus, Medline (via Web of Science), and EMBASE (File S1).

**FIGURE 1:**
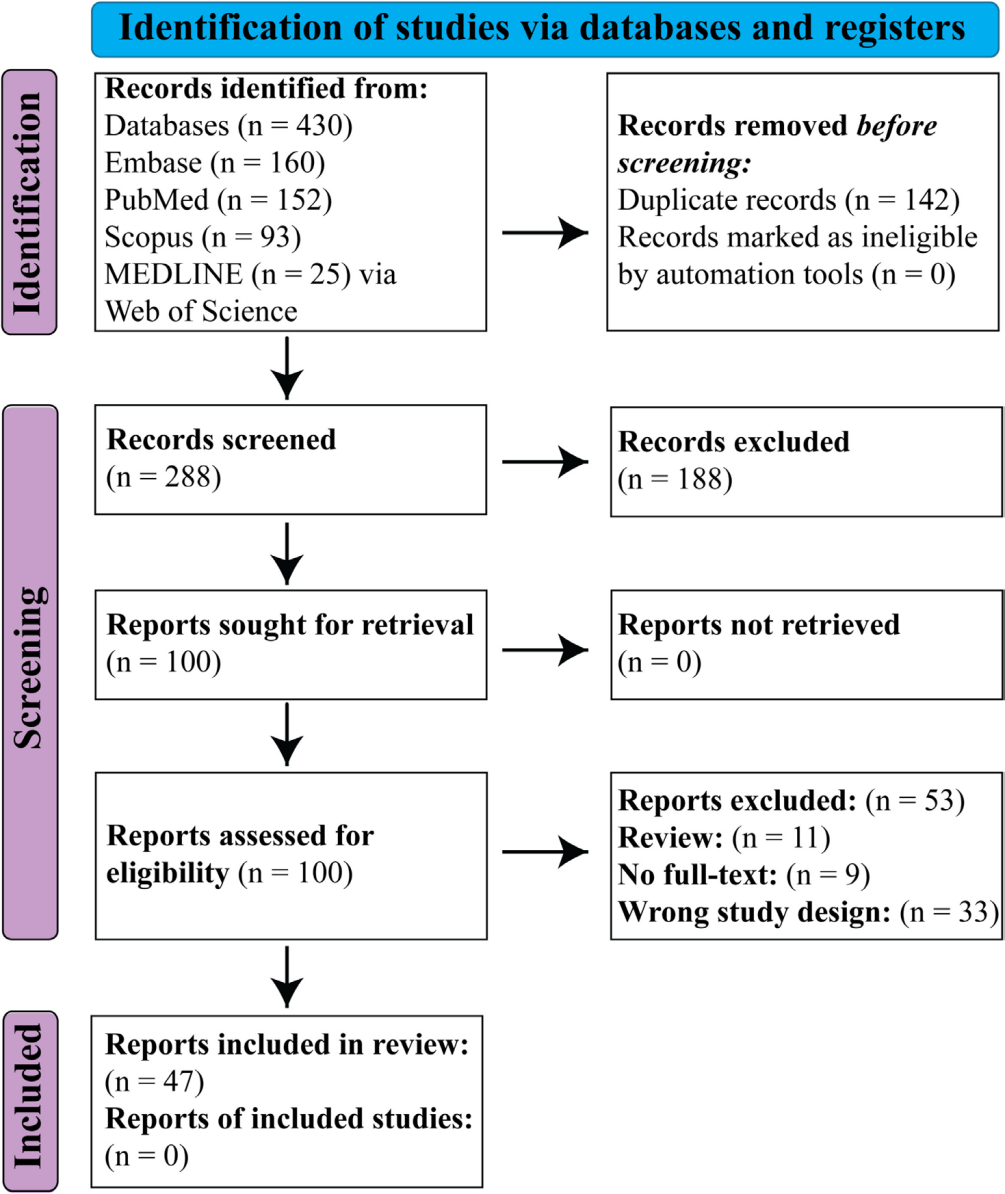
PRISMA flow diagram for study selection process: A total of 430 records were identified across four databases (Embase, PubMed, Scopus, and MEDLINE). After removing 142 duplicates, 288 records were screened, and 188 were excluded. Full texts of 100 studies were assessed for eligibility, with 53 further excluded due to being reviews, lacking full text, or having the wrong study design. Ultimately, 47 studies were included in the review.

Two reviewers independently screened the titles and abstracts. The full articles were then assessed within the Covidence platform. Discrepancies were discussed and resolved by the reviewers. Review articles, articles without full text, and studies not evaluating the effectiveness of iron chelation on colon or rectal cancer were excluded. Original research articles specifically investigating the impact of iron chelation on colon or rectal cancers were included, resulting in 47 studies directly relevant to this review. Figure 1 represents the literature search methodology for this study, following PRISMA guidelines for systematic reviews (File S2). Data (Study design‐ in vivo or in vitro and different colon cancer cell lines used, animal model used, category of the iron chelator used, IC50, results and the mechanism related to iron chelation) were extracted from the selected articles by two independent reviewers.

## 3 Results and Discussion

### 3.1 Iron Metabolism in CRC

Iron levels in normal cells are tightly regulated by iron regulatory proteins to prevent iron‐induced cytotoxicity [10]. The balance between iron surplus and deficiency plays a crucial role in tumorigenesis, progression, metastasis, and treatment response [9]. Emerging evidence suggests that iron plays a dual role in patients with CRC. High iron intake, particularly heme, is linked to increased CRC risk and progression [8, 33, 34]. Iron deficiency can suppress CRC growth through reduced immune surveillance [8]. One of the essential hallmarks of cancer is the rapid proliferation and uncontrolled division, which significantly increases the synthesis of nucleic acids and proteins, thus elevating the energy demand [35]. To support this, cancer cells require substantial iron, which is supplied by modulating iron acquisition, trafficking, and storage [36]. Many studies have revealed an upregulation of iron uptake and storage proteins in CRC tissues compared to non‐neoplastic adjacent colon tissues [14, 36, 37].

Alterations in iron metabolism are a critical aspect of CRC progression. Several proteins involved in iron import, such as transferrin receptor 1 (TFR1), DMT1, duodenal cytochrome B ferrireductase (TCYBC), and heme‐oxygenase 1(HO‐1), have been identified as upregulated in CRC tissues and cell lines [14, 38]. CRC cells exhibit elevated levels of TFR1 to increase transferrin‐bound iron uptake, as well as lipocalin 2 (LCN2), a siderophore‐binding protein that further aids in iron acquisition [8, 14, 39]. Additionally, CRC cells express higher levels of DMT1 to increase the labile iron pool [8].

**FIGURE 2:**
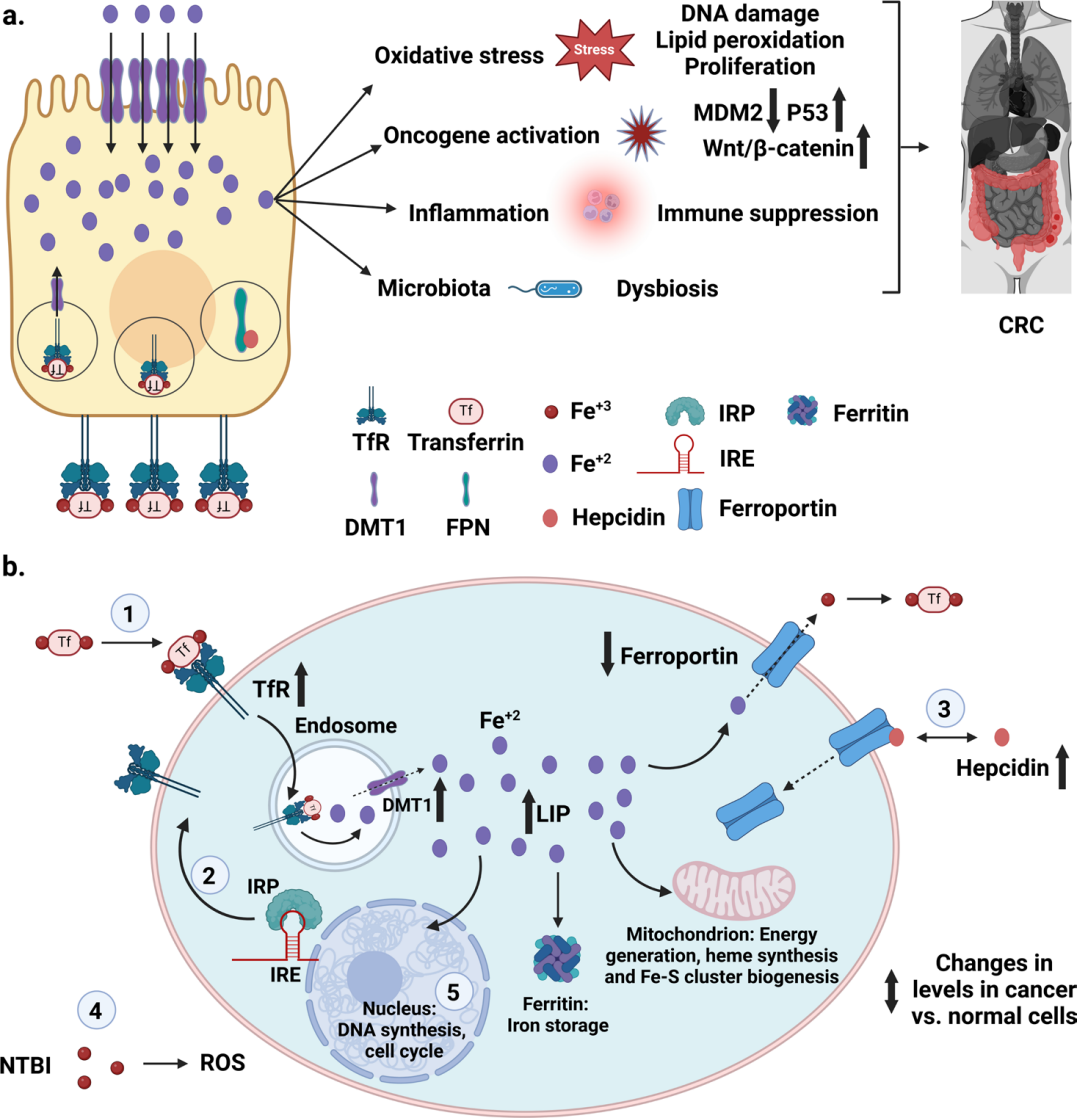
Iron metabolism in CRC pathogenesis and progression. (a) Iron‐mediated CRC progression. High intracellular iron levels in the tumor cells lead to increased reactive oxygen species (ROS) synthesis, oncogene activation, pro‐inflammatory mediators, and dysbiosis. Reproduced via Biorender from Ref [36] with permission under a Creative Commons Attribution Licence (CC‐BY). (b) Iron metabolism in CRC. Transferrin receptors (TfR), labile iron pool (LIP), iron‐regulatory proteins (IRP), iron‐response elements (IRE), non‐transferrin‐bound iron (NTBI). (1) Transferrin‐bound iron binds to the plasma membrane's transferrin receptors (TfR). (2) Iron metabolism is controlled by iron‐regulatory proteins (IRP) at the cellular level. (3) Hepcidin is the main iron regulatory hormone that maintains systemic iron homeostasis. (4) High systemic iron levels can lead to Tf saturation. Hence, non‐transferrin‐bound iron (NTBI) formation follows. The uptake of NTBI can lead to the formation of reactive oxygen species (ROS), resulting in oxidative stress and cell damage. (5) In cancer cells, genes encoding proteins that increase intracellular iron (TfR, DMT1, hepcidin) are generally upregulated, whilst those decreasing iron levels (ferroportin) are downregulated. Reproduced via Biorender from Ref [46] with permission from Elsevier copyright [2014].

Another central hallmark in CRC is the involvement of Haber‐Weiss and Fenton‐type reactions, which are significant sources of oxidative stress. The Fenton reaction occurs when ferrous iron (Fe^2+^) and hydrogen peroxide (H_2_O_2_) react to produce ferric iron (Fe^3+^) and hydroxyl radical (OH.) [40]. The Haber‐Weiss reaction is a cascade of reactions that generates reactive oxygen species (ROS) [41]. In this process, hydroxyl radicals react with H_2_O_2_ to form more hydroxyl radicals (OH.) and hydroxyl anions (OH^−^). Nitric oxide (NO) also interacts with heme iron in some proteins, such as soluble Guanylate Cyclase (sGC) and cytochrome C [42]. Reversible binding of NO to iron in sGC activates several signaling pathways. Binding of NO to cytochrome C inhibits cellular respiration under hypoxic conditions. Additionally, NO can disrupt the iron–sulfur clusters, affecting the enzyme activities [42]. The interaction between iron and NO also plays a role in cancer pathogenesis by modulating iron metabolism, mitochondrial function, and oxidative stress. ROS homeostasis is critical for cellular processes. Low levels of ROS are essential for regulating cellular signaling cascades, while higher concentrations can initiate apoptosis [43, 44]. Increased ROS levels are directly associated with carcinogenesis in CRC, damaging proteins, lipids, and chromosomes [44]. It is speculated that high iron concentrations in colon cancer cells can induce oxidative stress, lipid peroxidation, and alterations in the cell cycle, all of which contribute to tumorigenesis [45]. Thus, understanding iron metabolic processes (Figure 2) and their effects on colorectal cancer is essential for elucidating their roles in CRC pathophysiology. This knowledge can inform the development of treatment strategies targeting iron metabolism in patients with CRC.

### 3.2 Different Categories of Iron Chelators Used in CRC

As iron is essential for the growth and proliferation of colon cancer cells, iron chelation is expected to inhibit the growth of these cancer cells. Targeting iron depletion in cells through chelation is emerging as a promising approach to cancer therapy [10]. Iron chelators can remove iron from cancer cells by forming complexes or binding directly to iron‐attached molecules. Several natural and synthetic iron chelators with different efficacies have been studied against CRC. These chelators can alter signaling pathways, such as the Wnt/β‐catenin pathway, the ROCK1/pMLC2 pathway, the histone methylation pathway, and the autophagy pathway. Additionally, they can modify the expression of several proteins such as p53, NDRG1, and cyclins. The alterations in protein expression and signaling pathways induced by iron chelators significantly impact the proliferation, invasion, and migration of colon cancer cells.

Numerous in vitro and in vivo studies have evaluated the effects of iron chelation in the tumor microenvironment on the proliferation and progression of CRC cells. Several iron chelators, including deferoxamine, Deferasirox, 4, 4‐dimethyl‐3‐thiosemicarbazone (Dp44mT), thiosemicarbazone chelators, polyaminocarboxylate‐based chelators, quilamine‐based chelators, novel synthetic chelators, and natural plant extracts, have been investigated for their potential against CRC (Table 2 and Table 3).

**TABLE 2:** Iron chelators used in the treatment of CRC ‐ Experimental findings.

Category of iron chelators	In vitro (cell lines used)	In vivo (animal model)	Main findings	Outcome (positive/negative)	IC50	Mechanism	Ref.
Deferoxamine (DFO)	HCT116 LoVo	NA	The proliferation of HCT116 and LoVo cells was inhibited significantly by the DFO treatment DFO‐induced global histone methylation changes in CRC cells	Positive	NA	Histone methylation changes	[18]
	HCT116 SW480 DLD1 RKO Patient‐derived enteroids	Mice	Treatment with DFO led to an average reduction in cell growth in all four CRC cell lines assessed	Positive	~10 μM	Maintaining nucleotide metabolism is the basic mechanism by which local iron handling is modulated	[47]
	HCT116	Mice	Iron depletion suppressed growth and tumorigenicity of human colon carcinoma cells in a p53‐dependent manner	Positive	NA	Iron depletion induces cell‐cycle arrest and suppresses tumor formation and cell proliferation in a p53‐dependent manner Tumor suppressor p53 protein is downregulated in iron excess	[48]
	SW480	NA	In vitro cytotoxicity experiments in 2D and 3D cancer cell cultures revealed moderate activity of the new complexes in SW480 with other prodrugs of carboplatin	Positive	Platinum(iv)–DFO complexes A‐168 ± 9 μM B‐215 ± 46 C‐ 255 ± 19 D‐78 ± 6 E‐89 ± 22 After 96 h	NA	[49]
	HT‐29 SW948	NA	There was an increase in the inhibition of the cancer cell growth when DFO was used with BmAbs	Positive	1.9 ± 0.3 μg/mL DFO	NA	[50]
	Epithelial cells from the normal human colon tissues HCT116 SW480 MC38	Mice	The combination of iron chelators and DNA‐damaging agents enhances DNA damage response and reduces colon tumor cell growth	Positive	NA	Downregulation of 𝛽‐catenin target gene c‐Myc reduced the transcription factor E2F1 and its target protein POLD1 Modulating TNKS/Axin2/𝛽‐catenin/c‐Myc/E2F1/POLD1 axis	[51]

	SW948 HT‐29	NA	Adding DFO enhanced the inhibitory activity of 1A10 and effectively prevented the regrowth of tumor cells in vitro	Positive	SW948 1.33 nM HT‐29 0.13 nM	NA	[52]
	HT29	NA	CE, gallic acid (GA) and DFO significantly reduced the number of human colon cancer HT29 cells	Positive	NA	Cancer cell number is reduced by CE and GA via a mechanism other than iron chelation	[53]
	HCT‐116 HT29 SW480	NA	Pretreatment of HCT116 cells with either DFO reduced the extent of DNA damage caused by EGCG	Negative	NA	NA	[54]
HCT116	NA	DFO treatment inhibited TRAIL‐induced cytotoxicity in HCT116 colon cancer cells, showing proliferative and protective effects of DFO on cancer cells	Negative	NA	DFO‐induced autophagy flux inhibited the TRAIL‐mediated anticancer effect	[21]
4, 4‐dimethyl‐3 thiosemicarbazone (Dp44mT)	HCT116 SW480	NA	Dp44mT induced over‐expression of NDRG1, which mediates cell viability, migration, and invasion and caused apoptosis of colon cancer cells	Positive	NA	Up‐regulation of NDRG1, which reduces the transforming growth factor beta‐1 (TGF‐β1) induced epithelial‐to‐mesenchymal transition (EMT) through activation of Wnt/β‐catenin signaling pathway	[55]
	HT29	NA	Dp44mT‐NPs were toxic towards colorectal HT29 cells	Positive	~14.5 μM—After 48 h	NA	[56]
	HT29 HCT116	NA	Cellular iron depletion by novel thiosemicarbazone iron chelators (e.g., di‐2‐pyridylketone 4,4‐dimethyl‐3‐thiosemicarbazone) induced NDRG1 up‐regulation NDRG1 overexpression inhibited cell migration	Positive	NA	Inhibition of the ROCK1/pMLC2 pathway via upregulation of NDRG1	[57]
Other Thiosemicarbazone chelators							
Thiosemicarbazones based on di‐2‐pyridine ketone and quinoline moiety.	HCT116	NA	All the compounds that were synthesized showed cytotoxic activity against the HCT 116 cells	Positive	Different IC50 values for 10 different compounds 0.00110 ± 0.00006–18.31 ± 0.92 μM	p53‐independent mechanism of apoptosis?	[58]
Bp4eT and its phase I metabolites	HCT116	NA	Bp4eT showed significant cytotoxic effects against HCT116 cells	Positive	Bp4eT—0.008 ± 0.001 μM	Suppression of the autophagy pathway by Bp4eT may make cells more susceptible to death induced by apoptosis and/or necrosis	[59]
(hetero)aromatic thiosemicarbazones	HCT‐116	NA	Several of the tested compounds showed submicromolar antiproliferative activity in HCT116 Compound 4b was one of the most active against HCT‐116 cells	Positive	Compound 3a—41.5 ± 1.7 μmol/L Compound 4b—4.86 ± 1.48 μmol/L	NA	[60]
Albumin Conjugates of Thiosemicarbazone and Imidazole‐2‐thione Prochelators	Caco‐2 HT‐29	NA	These chelators have antiproliferative activities at sub‐micromolar levels in the colon cancer cell lines	Positive	244mTC‐S for Caco‐2 0.38 ± 0.06 μM 244mTC‐S for HT‐29—0.9 ± 0.2 (IT1‐S) for Caco‐2 0.37 ± 0.02 μM (IT1‐S) for HT‐29—0.85 ± 0.15 μM After 72 h	NA	[61]
Combination of Fe/Cu‐chelators and docosahexaenoic acid	HCT116 HCT8/5‐FU	Mice	The triple combination of 5‐FU, DTN and DHA resulted in elevated apoptosis in CRC cells reducing the tumor size and weight in vivo and in vitro	Positive	NA	An increase of ROS and Mcl‐1 (Bcl‐2 family protein, inhibits apoptosis) ubiquitination	[62]
Polyaminocarboxylate based chelators	HT29	NA	The polyaminocarboxylate chelators NETA, NE3TA, and NE3TA‐Bn induced antiproliferative activity in HT‐29	Positive	NETA 7.3 ± 1.5 (μM) NE3TA 4.7 ± 0.3 NE3TA‐Bn 2.6 ± 0.2	NA	[63]
	HT29	NA	Bile acid–polyaminocarboxylate conjugates containing NE3TA are a potential iron chelator that displayed significant cytotoxicity in HT29 colon cancer cells	Positive	7.5 ± 0.3 μM	NA	[64]
	HT29	NA	N‐NE3TA–Tf conjugate displayed significant inhibitory activity against colon cancer cell lines Triazacyclononane‐based polyaminocarboxylate chelator NE3TA 7 showed significantly higher antiproliferative activity against colon cancer cells	Positive	N‐NE3TA–Tf—2.8 ± 0.1 μM N‐NE3TA—6.8 ± 0.4 μM	NA	[65]
Quilamine based chelators	Caco‐2 HCT‐116	Chinese Hamster Ovary cells	Polyaminoquinoline iron chelators, derived from the lead HQ1–44, Quilamines I have potential anticancer activity against HCT‐116 and Caco‐2 cell lines	Positive	HQ1–44‐ 1.3 μM HQ0–44 5 μM	NA	[66]
	HCT116	Xenografted nude mice	HQ1‐44 inhibited DNA synthesis and cell proliferation of HCT116 cells HQ1‐44 was as effective in reducing HCT116 tumor growth, without its side effects in xenografted athymic nude mice	Positive	Quilamine concentrations lower than 10 mM, the first component indicated the antiproliferative effect (62% of component 1 with IC50‐1 1 μM) For higher concentrations, the second component reflected the cytotoxic effect (38% of component 2 with IC50‐2165 μM)	By modulating the intracellular metabolism of both iron and polyamines	[67]
Other synthetic iron chelators							
[N,N′‐Bis(salicylidene)‐1,2‐phenylenediamine]metal complexes	HT‐29	NA	[N,N′‐bis(salicylidene)‐1,2‐phenylenediamine]iron(II/III) complexes 3 and 4 iron complexes are very effective cytotoxic agents against HT‐29	Positive	NA	Generation of intracellular ROS, which can induce apoptosis in cells, by the active compound, which contains a ligand with a large, conjugated π‐system and a potentially redox‐active iron centre Complex mode of action	[68]
Hydroxyphenyl hydrazone derivate YCL0426	SW480	NA	YCL0426 showed significant antiproliferative activity on cancer cell lines YCL0426 showed concentration‐dependent Fe (II) sequestering ability and induced upregulation of TfR1 and DMT1 expression	Positive	5.25 μmol/L	Inhibition of DNA synthesis, induction of G1/S phase cell cycle arrest, and induction of DNA damage were involved in its antiproliferative activity	[69]
Super‐polyphenol	HCT116	Xenograft model using BALB/c nude mice	SP6 and SP10 inhibited cancer cell proliferation by inducing apoptosis in HCT116 cancer cells SP10 also inhibited tumor growth in an HCT116 xenograft model SP6 and SP10 can chelate Fe3+ in a dose‐dependent manner	Positive	SP6–9.3–93.2 μg/mL SP10‐6.2–51.4 μg/mL	NA	[70]
3‐Chloro‐N′‐(2‐hydroxybenzylidene) benzohydrazide	HCT‐116 HT‐29	NA	CHBH, a derivative of PIH, has the iron chelating potential which enhances its anticancer activity CHBH reduces cancer cell proliferation by impairing iron metabolism and modulating chromatin structure via LSD1‐selective inhibition	Positive	HT‐29–0.95 μM—After 72 h	Chelation of iron from cancer cells via LSD1 inhibition, CHBH triggers wide‐ranging histone alterations responsible for the reactivation of tumor suppressor genes	[71]
Acyl Hydrocarbons	SW480 DLD‐1 SW620 HCT116	NA	Tested acyl hydrocarbons inhibited the growth of DLD‐1 and SW480 cells	Positive	0.5 to 1.7 mmol/L	Inhibition of Wnt signaling pathway	[72]
2,2^/^−dipyridyl	Lovo	NA	Hypoxia and iron chelator 2,2^/^−dipyridyl treatment can stimulate the invasion and migration enhancement of Lovo cells	Negative	NA	HIF‐1α protein expression and stabilization	[73]
Deferasirox	SW480 HCT116 DLD1	Mice	DOXjade inhibited tumor cell growth in a dose and time‐dependent manner in the human CRC cell lines	Positive	HCT‐ 36.42 μg/mL	Iron depletion‐induced TfR protein down‐regulation and apoptotic cell death under multimodal treatments	[74]
Alginate	RKO	NA	Alginate inhibited cellular iron transport through extracellular iron chelation with the resulting complexes not internalized	Positive	NA	Alginate inhibits cellular iron transport by binding the iron in solution, and the resulting complex remains extracellular	[75]

**TABLE 3:** Plant extraction‐based iron chelators used in the treatment of CRC ‐ Experimental findings.

Category of iron chelators	In vitro (cell lines used)	In vivo (animal model)	Main findings	Outcome (positive/negative)	IC50	Mechanism	Study
Epigallocatechin‐3‐gallate (EGCG)	HT‐29	NA	EGCG induced iron chelation activity, which inhibited the growth of HT‐29	Positive	EGCG–88.1 μM—After 72 h	NA	[76]
	RKO HCT116	Mice	EGCG inhibited cell proliferation and induced apoptosis EGCG suppressed angiogenesis and induced apoptosis in liver metastases without associated body weight loss or hepatotoxicity in SCID mice	Positive	NA	Apoptosis in human CRC through Akt, p38 and VEGFR2, and VEGFR2 suppresses angiogenesis and liver metastases of CRC	[77]
	HT‐29	NA	Apoptosis was induced in HT‐29 cells by ER (Endoplasmic reticulum) stress after the EGCG treatment	Positive	262.5 μM—After 24 h 190.3 μM—After 48 h 88.1 μM—After 72 h	ER stress is induced by EGCG and activates UPR proteins, PERK (with its downstream targets eIF2α and ATF4), and IRE1α	[78]
Green Tea plant extract	HCT‐116 SW480	NA	Most of the tested tea polyphenols showed dose‐dependent antiproliferative effects, and EGCG showed the most potent antiproliferative activities against CRC cells	Positive	NA	Cell cycle arrest in the G1 and apoptosis induction	[79]
Hairy roots of turnip *Brassica rapa ssp. rapa*	HT‐29	NA	Antioxidant activity and ferrous ion chelating ability assays were significantly higher in hairy roots Hairy roots and non‐transformed root extracts tested against the HT‐29 cell line resulted in antiproliferative activity	Positive	NA	The chelating agent may inhibit radical generation by stabilizing transition metals, thus reducing free radical damage	[80]
*Peucedanum chenur* chloroformic extract (PCCE)	HCT116	NA	PCCE has an antiproliferative function against HCT‐116 cells and iron chelating ability	Positive	120 μg/mL—After 24 h 110 μg/mL—After 48 h 80 μg/mL—After 72 h	Suppressive effects on the Wnt/β signalling pathway by increasing the expression of the APC gene	[81]
Ethyl acetate and ethanol extracts from *D. simplex* flowers	Caco‐2	Mice	Ethyl acetate extract, which contains the highest flavonoid content, showed stronger metal (Fe^2+^) chelating activity *D. simplex* flowers showed significant antioxidant potential associated with important anti‐inflammatory capacity and anti‐proliferative effect against Caco‐2 cancer cells	Positive	Ethanol extract of *D. simplex* flowers—62.0 ± 0.5 μg/mL Ethyl acetate extract of *D. simplex* flowers—63.25 ± 0.25 μg/mL	NA	[82]
Aqueous root extract (ARE) of *H. angustifolia L* and ethanolic root extract (ERE)	DLD‐1	Female BALB/c nude mice	ERE showed a higher iron chelating ability than ARE ARE exhibited a stronger cytotoxic effect than ERE against human colon cancer DLD‐1 cells In vivo study demonstrated that 200 mg/kg/d of ARE administration could significantly and effectively inhibit tumor growth in tumor xenografts bearing BALB/c nude mice	Positive	ARE‐ 82.31 ± 9.62 μg/mL ERE‐ 89.18 ± 8 μg/mL	NA	[83]
Methanolic extracts from *Citrus aurantium L*. leaves at two development stages (young and old leaves)	DLD‐1	NA	Old leaf extract (OLE) showed a high capacity to chelate ferrous ions in comparison to young leaf extract (YLE) OLE revealed higher cytotoxic activity against DLD‐1 cells than the YLE Young and old leaves may be a source of natural protective agents against oxidative damage and possess anticancer properties	Positive	OLE‐IC50_resazurin_ = 7.3 and IC50 _Hoechst_ = 7.8 μg/mL YLE (IC50_resazurin_) = 22 and IC50_Hoechst_ = 24 μg/mL	NA	[84]
Extracts prepared from peel and seeds of melon ( *Cucumis melo L. reticulatus* group)	HT29	NA	Significant amounts of gallic acid, catechin, and eugenol were found in all the extracts of melon peel Different extracts of melon showed iron ion chelating activity at different concentrations All extracts and all concentrations tested exhibited antiproliferative activity below 50% in HT29	Positive	Extract of melon peel—4.0 mg/mL for Melon seed extracts—2.8 mg/mL	NA	[85]
Different fractions from the bark of *C. pictus*	HT29	NA	The chloroform fraction of the bark of *C. pistus* showed a concentration‐dependent metal chelating activity The viability of HT29 cells was significantly decreased in a dose‐dependent manner after treatment with different fractions of *C. pictus*	Positive	Chloroform fraction of bark–125 μg/mL, after 24 h	NA	[86]
Seed coat extracts of pea (*Pisum sativum L*.)	LS174	NA	Pea seed coat extracts 1–5 (Aslaug, Assas, Dora, Golf, Poneka), 9 (MBK 168), and 10 (MBK 173) concentration‐dependent cytotoxic actions against on colon adenocarcinoma LS174 Epigallocatechin and gallic acid were the two compounds with the highest in vitro antioxidant activity of pea seed coat extracts	Positive	Aslaug‐ 1.97 ± 0.51 μM Assas‐ 2.39 ± 0.17 μM Dora‐ 3.36 ± 0.81 μM Golf‐ 5.48 ± 0.52 μM Poneka‐ 2.90 ± 0.45 μM MBK 168–1.84 ± 0.26 μM MBK 173–2.41 ± 0.55 μM	Caspase‐3 dependent apoptosis + Other mechanisms	[87]
Essential oil isolated from the leaves of *Toddalia asiatica (L.) Lam*	HT‐29	NA	There was cytotoxicity of oil against colon (HT‐29) cancer cells; the extracted oil showed potent iron‐chelating activity	Positive	100.0 ± 0.16 μg/mL	NA	[88]
Aqueous extract of *Equisetum arvense L*	HT‐29 HCT‐8‐(HRT‐18) HCT 116	NA	There were significant iron‐chelating properties and cytotoxic properties of *Equisetum arvense L* against human colorectal carcinoma cell lines without any cytotoxicity towards the normal (HUVEC) cell line	Positive	HCT‐8 [HRT‐18]‐ 584 μg/mL HCT 116–341 μg/mL HT‐29‐ 337 μg/mL	NA	[89]
Methanolic extract of *Peucedanum chenur*	HCT116	NA	*P. chenur* methanolic extract increased apoptosis and reduced cell migration significantly *P. chenur* methanolic extract has iron chelating property	Positive	182.1 μg/ml—After 48 h 163.2 μg/mL—After 72 h	Induction of apoptosis through mitochondrial pathway, as increased BAX/BCL‐2 and inhibition of invasion through reduction of AKt1, FAK, RhoA, and MMP‐13 expression	[90]

#### 3.2.1 Deferoxamine (DFO)

Deferoxamine (DFO), the first iron chelator investigated as an anticancer agent, has been shown to inhibit the growth of multiple colon cancer cell lines, including HCT116, Lovo, SW480, and HT‐29, among others [18, 47, 49, 50, 51, 52, 53, 54]. Direct transfection of these colon cell lines with DFO has significantly reduced cancer cell proliferation, suggesting a strong relationship between iron availability and CRC progression [49, 50, 51, 52]. While DFO has been assessed in a phase I clinical trial for hepatocellular carcinoma and in a phase II trial for neuroblastoma, demonstrating antiproliferative effects [91, 92], no clinical trials have yet been conducted in CRC patients. Moreover, DFO has been used as a chelating agent in some studies to investigate the critical role of intracellular iron accumulation in CRC development.

Transferrin receptors, located on the cell surface, facilitate iron uptake and are overexpressed in CRC cells to increase iron availability for essential functions such as nucleotide synthesis, DNA repair, and cell proliferation [51, 93]. Several in vivo studies have demonstrated that TfR disruption can inhibit cancer cell growth by limiting iron availability within the tumor microenvironment [51]. However, the mechanism is complex and involves multiple signaling pathways. Iron is essential for the activity of tankyrase (TNKS), a metal‐dependent enzyme that degrades Axin2 and subsequently activates the β‐catenin/c‐Myc/E2F1/POLD signaling pathway [51]. Iron deficiency can cause the reduction of TNKS activity, leading to increased β‐catenin phosphorylation, oxidative stress, DNA damage, and apoptosis [51].

Additionally, patient‐derived colonoids treated with DFO have shown reduced gene expression for cyclin‐dependent kinase 1 (CDK1), which promotes cell proliferation through the phosphorylation of key signaling proteins, and POLD1, a gene associated with cancer cell progression and metastasis [47]. Some studies have also explored DFO as a combined treatment with contemporary anti‐cancer drugs such as carboplatin, as well as with DNA‐damaging agents or antibodies targeting cancer cells [49, 50, 51, 52]. Notably, DFO has been shown to enhance the antiproliferative effects of these agents. Harringer S. et al. reported moderate antiproliferative activity when DFO was combined with carboplatin in both 2D and 3D cell culture models of SW480 cells [49].

DFO has been shown to induce changes in the gene expression profile and histone methylation of colon cancer cells. Treatment with DFO has led to both upregulation and downregulation of numerous genes in CRC cells [18]. Functionally, these gene expression changes have impacted processes such as cell cycle maintenance, apoptosis, and signaling pathways involved in cell growth. Interestingly, combined with certain natural plant extracts has shown significant inhibition of colon cancer cell growth [53]; however, in some cases, the combination has reduced the antiproliferative effect of the plant extract alone [54]. These mixed outcomes may be attributed to the complex chemical composition of the plant extracts. In vivo studies also support the antiproliferative effects of DFO, demonstrating reduced tumor growth and CRC tumor size in murine models [47, 48].

The half maximal inhibitory concentration (IC50) is a key measure of drug efficacy, indicating the concentration required to inhibit a biological process by 50%. Lower IC50 values indicate a drug's effectiveness at lower concentrations, potentially minimizing systemic toxicity during treatment. Studies investigating the IC50 values of DFO for treating colon cancer cell lines have shown that, particularly when combined with other agents such as antibodies or drugs, DFO exhibits varied IC50 values, reflecting additive, synergistic, or antagonistic effects [49, 50, 52].

While most studies highlight the anticancer potential of DFO, a few report its potential negative effects. For instance, Moon et al. observed that DFO could exert a proliferative and protective effect on colon cancer cells [21]. Tumor necrosis factor (TNF)‐related apoptosis‐inducing ligand (TRAIL) mediated apoptosis, a potential CRC therapeutic mechanism, was inhibited by DFO through modulation of autophagy in CRC [21]. Additionally, Hu et al. showed that hypoxia‐induced autophagy could promote cancer cell growth in neuroblastoma [94]. These findings suggest that while DFO can exert anticancer effects in CRC, in vitro and in vivo studies reveal a complex profile, with some reports indicating that DFO‐induced iron deficiency may promote CRC growth.

A notable gap in many studies is the lack of focus on intracellular iron levels following treatment with iron chelators, such as DFO. Measuring iron concentrations after treatment is essential to understand DFO's anticancer mechanism better. A study examining the effects of DFO‐induced iron deficiency in breast cancer cell lines found a decrease in mitochondrial iron concentration in MCF‐7 cells, while in MDA‐MB‐231 cells, mitochondrial iron concentration increased post‐treatment [22]. Overall, DFO demonstrates potential as an anticancer agent in CRC through iron chelation and modulation of gene expression, though its effects are complex and context‐dependent, highlighting the need for further investigation into its mechanisms and impact on intracellular iron distribution.

#### 3.2.2 Thiosemicarbazones

Thiosemicarbazones represent another category of iron chelators that have been used to induce antiproliferative effects in CRC cells. Dp44mT is one such thiosemicarbazone tested against CRC cells for antiproliferative effects [55, 56]. It has shown positive outcomes in reducing cell migration and invasion, which has a direct effect on metastasis [55]. Flow cytometry assays indicate that Dp44mT can inhibit the metastatic potential of certain colon cancer cell lines [55]. This compound promotes autophagy and apoptosis in CRC cells and suppresses epithelial‐to‐mesenchymal transition (EMT) [55]. The antiproliferative mechanism of Dp44mT has been linked to the overexpression of NDRG1, which activates the Wnt/β‐catenin signaling pathway, resulting in a decrease in transforming growth factor beta‐1 (TGF‐β1) expression, a key factor in metastasis [55]. Additionally, several newly synthesized thiosemicarbazone‐derived iron chelators have shown cytotoxic effects in CRC [58, 59, 60, 61, 62]. Proposed mechanisms for these novel chelators include autophagy suppression, inhibition of the ROCK1/pMLC2 pathway, and induction of p53‐independent apoptosis, often via upregulation of NDRG1 [57]. The IC50 values of these thiosemicarbazone‐based chelators range from 0.008 to ~14.5 μM, though not all studies confirmed the IC50 values as indicators of cytotoxic efficacy [56, 58, 59]. Taken together, thiosemicarbazones, such as Dp44mT, show promise in CRC treatment by inhibiting metastasis and promoting cell death through mechanisms like Wnt/β‐catenin pathway activation and autophagy suppression.

#### 3.2.3 Polyaminocarboxylate‐Based Chelators

Various polyaminocarboxylate‐based chelators have been evaluated for their antiproliferative effect against CRC cells [63, 64, 65]. Bile acid was conjugated with these polyaminocarboxylate chelators to enhance the pharmacokinetic properties, facilitating the identification of colon cells that produce bile acid transporters, carriers, and receptor proteins [64]. This approach enables targeted iron depletion therapy in CRC. Bile acid–NE3TA conjugates represent some of the first targeted iron‐depleting agents studied in CRC. Furthermore, combining polyaminocarboxylate‐based iron chelation therapy with sensitive cancer imaging techniques, known as theranostics, has yielded promising results [65]. These theranostic strategies allow for the integration of therapeutic options with imaging modalities. A study has demonstrated anticancer effects against CRC using theranostics that combine a polyaminocarboxylate‐based iron chelator with near‐infrared (NIR) fluorescent transferrin conjugates [65]. The reported IC50 values for these chelators range from 2.6–7.4 μM [63, 64, 65]. Despite their significant antiproliferative effects, the underlying mechanisms of these polyaminocarboxylate‐based iron chelator analogues remain inadequately explored in the current literature.

#### 3.2.4 Quilamine‐Based Iron Chelators

New generations of quilamine‐based iron chelators have shown anticancer effects against CRC. HQ1–44, a first‐generation quilamine with high affinity, demonstrates significant anticancer properties [66, 67]. Derived from HQ1–44, new polyaminoquinoline compounds exhibit potent effects against the HCT116 cell line, originating from human colon adenocarcinoma and can arrest certain cell cycle phases in CRC cells [66, 67]. Modified quilamine‐based chelators display variable IC50 values, and in vivo studies have confirmed reduced tumor burden in xenografted nude mice with CRC [66, 67]. Mechanistically, these chelators inhibit DNA synthesis and induce cell cycle arrest, leading to apoptosis and notable antiproliferative effects [67].

#### 3.2.5 Other Synthetic Iron Chelators

Several novel iron chelators have been developed and investigated as potential therapeutic candidates for CRC. N, N′‐Bis(salicylidene)‐1,2‐phenylenediamine, an iron chelator, demonstrates cytotoxicity against HT‐29 cancer cells, primarily through reactive oxygen species generation and apoptosis induction [68]. Another novel iron chelator, YCL0426, derived from hydroxyphenyl hydrazone, has a significant antiproliferative effect on CRC, with an IC50 value of 5.25 μmol/L [69]. YCL0426 induces G1/S cell cycle arrest, DNA damage, and inhibits DNA synthesis, contributing to its antiproliferative activity [69]. Acyl hydrazone chelators have also shown effectiveness against DLD‐1 and SW480 cell lines, with IC50 values ranging from 0.5 to 1.7 mmol/L [72]. Their mechanism of action appears to involve alterations in the Wnt/β‐catenin signaling pathway, contributing to their antiproliferative effects [72].

Additionally, super polyphenols (SPs) are another novel candidate for iron chelation therapy in CRC. Specifically, SP6 (IC50‐9.3–93.2 μg/mL) and SP10 (IC50‐6.2–51.4 μg/mL) inhibit cancer cell growth in vitro and in vivo by inducing apoptosis [70]. Alternatively, pyridoxal isonicotinoyl hydrazone (PIH), a tridentate ligand, can sequester cellular iron and inhibit DNA synthesis, thereby exerting antiproliferative effects in CRC [71]. Certain PIH derivatives, such as 3‐chloro‐N‐(2‐hydroxybenzylidene) and benzohydrazide (CHBH), have shown positive therapeutic outcomes in CRC without affecting normal colon cells. This selectivity may be attributed to chromatin modulation and histone alterations [71]. Furthermore, alginates—a diverse group of biomolecules derived from brown algae—have demonstrated iron‐chelating potential. Alginates effectively bind with iron, inhibiting its cellular transport, which makes them promising candidates for CRC treatment, especially in cases where excessive iron is implicated in disease pathogenesis.

A diverse range of novel iron chelators, including quilamines and synthetic compounds, have shown promise in CRC treatment by inhibiting cancer cell proliferation through mechanisms like DNA synthesis inhibition, cell cycle arrest, and apoptosis, though some chelators may enhance invasive properties, highlighting the importance of selective targeting in therapy. However, 2,2′‐dipyridyl (DP), a lipid‐soluble iron chelator, has been observed to stimulate the invasion and migration of Lovo cell lines with an IC50 of 0.95 μM [73]. This effect is attributed to DP's role in stabilizing and activating HIF‐α, which promotes cell migration and invasion [73].

#### 3.2.6 Natural Iron Chelators in CRC

Recent interest in natural plant extracts as a cancer therapy has led to research into their potential role as iron chelators in CRC, although the results remain debated [95]. Various natural plant extracts with potential iron chelation properties were studied for their anticancer effects in CRC (Table 3). Epigallocatechin Gallate (EGCG), the most abundant component in green tea, exhibits a high affinity for iron, thereby enhancing its potential as an iron chelator [76, 77, 78, 79]. EGCG's iron‐chelating activity contributes to its antiproliferative effects through mechanisms like G1 cell cycle arrest, endoplasmic reticulum stress, and the modulation of oncogenic signaling pathways, all promoting apoptosis in CRC [77, 78]. Several other plant extracts have demonstrated antiproliferative effects in CRC through iron chelation, including hairy roots of turnip *Brassica rapa ssp rapa* [80], *Peucedanum chenur* [81], *D. simplex* flowers [82], *H. angustifolia L* roots [83], *Citrus aurantium L*. leaves [84], peel and seeds of melon ( *Cucumis melo L. reticulatus* group) [85], which have been experimented with as potential anticancer agents against CRC. Moreover, the bark of *C. pictus* , essential oil isolated from the leaves of *Toddalia asiatica (L.) Lam* [96], *Equisetum arvense L*. [89] and *Peucedanum chenur* [90] are some other natural plant extracts with iron chelation properties and antiproliferative effects in CRC.

Most studies report IC50 values for the plant extracts used. EGCG, one of the most widely studied extracts, demonstrated an IC50 of 88.1 μM after 72 h of treatment in CRC cells [76, 78]. These findings suggest that herbal medications containing plant extracts with iron‐chelating properties may offer a viable adjunct to current CRC treatments.

### 3.3 Mechanisms Related to Antiproliferative Effect by Iron Chelation

**FIGURE 3:**
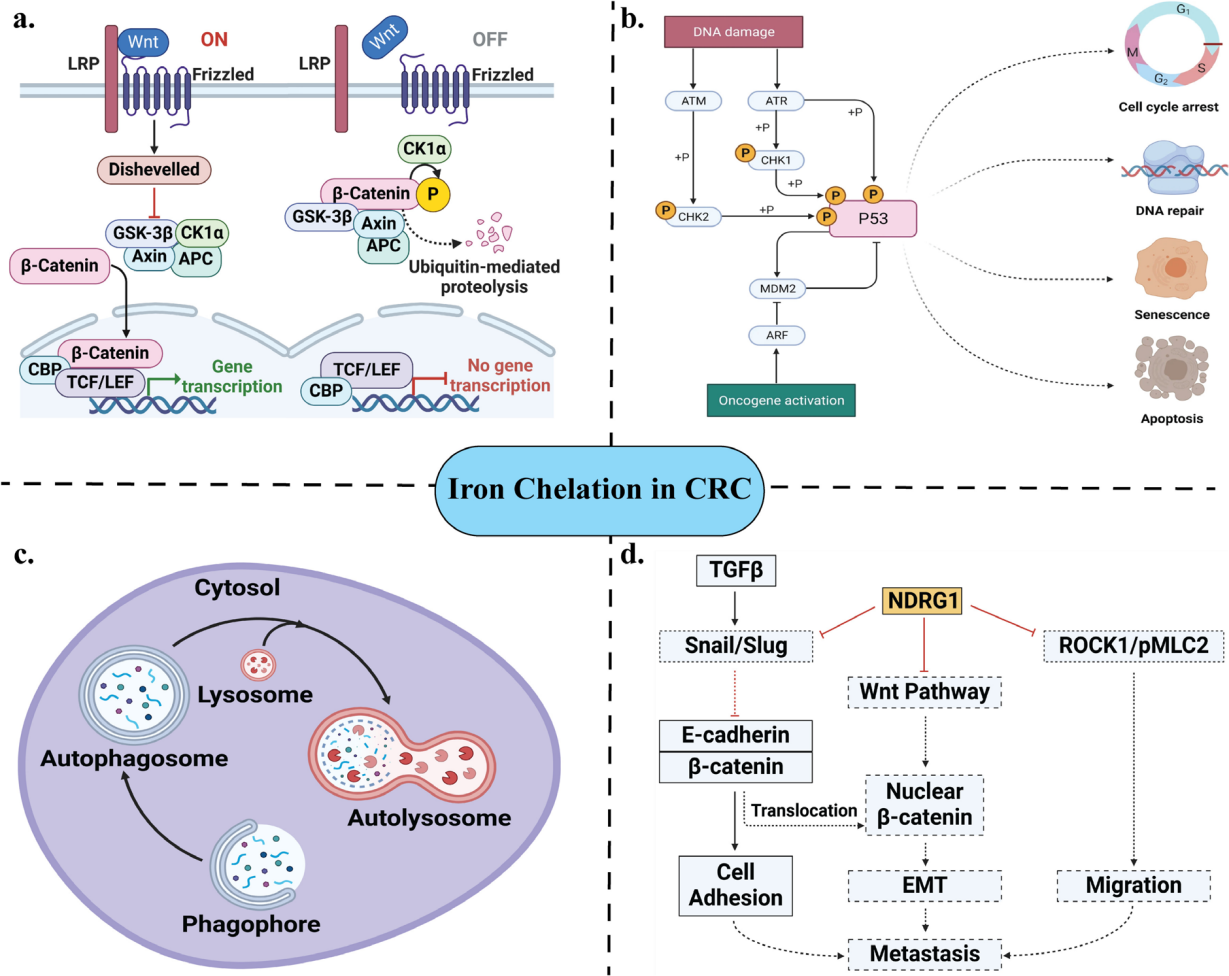
Schematic representations of the effects of iron chelation on CRC pathogenesis and progression. (a) Modulations in the Wnt/β‐catenin signaling pathway. Reproduced via Biorender from Ref. [100] with permission under a Creative Commons Attribution Licence (CC‐BY) copyright the authors[2020] (b) Upregulation of the p53 signaling pathway. Reproduced via Biorender from Ref [101] with permission under a Creative Commons Attribution Licence (CC‐BY) copyright the authors [2021]. (c) Alterations in autophagy (d) Upregulation of NDRG1. Reproduced via Biorender from Ref. [102] with permission copyright the authors [2013].

Studies consistently demonstrate that iron chelation has significant anticancer effects on CRC. High iron levels in CRC activate several iron‐dependent biochemical pathways, including Wnt signaling, p53 modulation, STAT3 pathways, and ROS generation [11]. Iron chelation disrupts these pathways, potentially reversing key CRC hallmarks such as uncontrolled proliferation, invasion, and metastasis [15]. Additionally, iron chelators activate ER stress signaling pathways leading to apoptosis [97]. According to Hanan and Weinberg, the six hallmarks of cancer include sustained proliferative signaling, apoptosis resistance, evasion of growth suppressors, angiogenesis, uncontrolled replication, and metastasis activation [98]. Iron chelators interfere with these hallmarks, positioning them as strong candidates for effective anti‐cancer therapy [99]. Key mechanisms underlying the anticancer effects of iron chelation in CRC include changes in histone methylation, modulations of the p53 signaling pathway, downregulation of Wnt/β‐catenin signaling pathway, upregulation of NDGR1, and alterations in autophagy (Figure 3).

#### 3.3.1 Modulating the Wnt/ β‐Catenin Signaling Pathway

Alterations in the Wnt/β‐catenin signaling pathway play a key role in cancer progression [103]. Iron chelation can modulate certain proteins associated with this pathway, including Wnt3a, β catenin, and Cyclin D1 [104]. The Wnt/β‐catenin pathway is organised into extracellular, membrane, cytoplasmic, and nuclear segments [103]. Wnt3a is involved in extracellular signaling, while β‐catenin operates within the cytoplasm. Dysregulation in the Wnt/β‐catenin signaling pathway plays a pivotal role in CRC progression, invasion, and metastasis, therapy resistance, and recurrences [105, 106].

In CRC, mutations are often observed in the adenomatous polyposis coli (APC) gene, which is a key tumor suppressor [107]. The *APC* gene encodes proteins with roles in chromosome segregation, microtubule assembly, cell adhesion, and migration [108]. Importantly, *APC* regulates the β‐catenin levels, which are central to CRC pathogenesis [107]. Also, *APC* gene mutations can activate β‐catenin, leading to changes in its phosphorylation sites. Furthermore, altered β‐catenin levels influence downstream targets such as MYC and Cyclin D1, both critical to the β‐catenin signaling pathway. Cyclin D regulates cell cycle progression from the G1 to S phase, while MYC and Cyclin D expression subsequently enhance epithelial cell proliferation, promoting tumor growth [109]. Iron chelation, particularly with DFO and acyl hydrazones, has been shown to attenuate the Wnt/β‐catenin signaling in colon cancer cell lines, potentially limiting tumor progression [51, 72].

#### 3.3.2 Upregulation of p53 Signaling Pathway

Iron depletion has been shown to suppress the growth and tumorigenicity of colon cancer cells in a p53‐dependent pathway [48, 58]. Dysregulation of the p53 tumor suppressor gene plays a significant role in CRC development, influencing traits such as invasiveness, metastasis, and prognosis [72, 110]. Excess iron in cells downregulates p53, potentially leading to tumor progression. In both in vitro and in vivo studies, iron chelation with DFO has been shown to inhibit CRC growth by stabilizing and reactivating p53 through a p53‐dependent mechanism [48]. Hence, iron chelation effectively stabilizes and activates p53, reinforcing its tumor‐suppressive role by promoting cell cycle arrest and apoptosis in CRC, particularly in cells with DNA damage. On the contrary, a study on hepatic carcinogenesis showed that DFO‐induced moderate iron deficiency increases MDM2 levels and subsequently decreases p53 levels [111].

#### 3.3.3 Upregulation of NDRG1

The role of NDRG1 (N‐myc downstream‐regulated gene 1) in iron chelation therapy has been widely investigated due to its association with cell proliferation, differentiation, development, adhesion, DNA repair, and response to cellular stress. It also regulates p53‐mediated caspase activation and apoptosis [112, 113]. NDRG1 is recognized as a metastatic suppressor protein, although its role in cancer varies, showing both tumor‐promoting and tumor‐suppressing effects depending on the context [114]. In CRC, NDRG1 has shown anti‐oncogenic properties by inhibiting cancer cell proliferation, epithelial‐to‐mesenchymal transition (EMT), metastasis, and migration [115, 116].

NDRG1 interacts with several key signaling pathways (e.g., snail/slug, Wnt, EMT) and molecules such as TGF‐β and EGFR, which are involved in tumor progression. EMT, a process by which epithelial cells acquire mesenchymal characteristics, is linked to metastasis in CRC and is regulated by factors like TGF‐β and transcriptional repressors Snail and Slug [117, 118]. The Wnt signaling also promotes metastasis by raising nuclear β‐catenin levels, while the ROCK1/pMLC2 pathway contributes by regulating actin filament assembly, which is in turn essential for cell movement [57].

Novel iron chelators such as Dp44mT and DpC upregulate NDRG1, which subsequently inhibits metastasis by disrupting Snail/slug, Wnt, and ROCK1/pMLC2 pathways [49, 50, 51, 52]. These chelators, particularly Dp44mT, counteract TGF‐β1‐induced EMT in colon cancer, thereby inhibiting cancer progression [55]. Additionally, Dp44mT modulates NDGR1 expression, suppressing CRC cell proliferation, invasion, and migration while inducing apoptosis [55].

NDRG1 also impacts several oncogenic pathways (e.g., STAT3, Wnt/β‐catenin, RAS, and AKT/PI3K), which contribute to the sustained proliferation and survival of cancer cells. Iron chelation attenuates these pathways, highlighting its potential as a multifaceted therapeutic approach for CRC [57, 102].

#### 3.3.4 Alterations in Autophagy

Autophagy is a conserved degradation process that removes damaged intracellular materials via lysosome‐dependent pathways, with both tumor‐suppressing and tumor‐promoting roles in cancer [119]. Early‐stage autophagy suppresses tumors by degrading oncogenic molecules, while in advanced cancer stages, it can promote tumor survival by supporting the cancerous microenvironment. Excess intracellular iron can generate ROS, inducing oxidative stress and, consequently, stress‐induced autophagy. However, the role of iron chelators in modulating autophagy remains poorly understood. A study on CRC found that a novel thiosemicarbazone‐based iron chelator suppressed autophagy and had antiproliferative effects [59]. Collectively, these findings suggest that while autophagy modulation holds potential in CRC treatment, further studies are needed to clarify how iron chelators may precisely influence autophagic pathways and their impact on tumor progression.

#### 3.3.5 Alterations in the Histone Methylation

Histone methylation regulates gene transcription, chromatin remodelling, cell cycle, and stress responses and is crucial in cancers, including CRC regulation, growth, and differentiation, DNA damage, and oxidative stress response [120]. In CRC, histone methylation changes contribute to tumor growth, cell migration, and silencing of tumor suppressor genes [121]. For instance, H3K9me3, a transcription repression marker, was found elevated in late‐stage CRC and is linked to enhanced tumor cell migration [122]. Moreover, enzymes involved in histone methylation, such as histone methyltransferases and demethylases, have also been implicated in CRC [121]. Consequently, targeting combined histone methylation modifications presents a promising therapeutic strategy for CRC management.

Recent evidence shows that iron chelation with DFO leads to increased levels of multiple histone methylations, including H3K4me2, H3K9me2, H3K9me3, H3K36me2, H3K36me3, H3K27me3, and H3K79me [18]. These histone methylations represent epigenetic modifications on histone proteins, which are crucial for DNA packaging and gene regulation. Notably, iron chelation influences these modifications without altering the abundance of histone methyltransferases and demethylases, indicating that iron chelation specifically impacts the catalytic activity of these enzymes rather than their expression levels [18]. Additionally, a novel iron chelator, a PIH derivative, has been reported to cause histone alterations that reactivate tumor suppressor genes [71]. This suggests that changes in histone methylation could be a mechanism through which iron chelators exert their antiproliferative effects in colon cancer cells.

#### 3.3.6 Cell Cycle Arrest Induced by the Iron Chelators

Inhibition of cell proliferation by iron chelators is closely associated with cell cycle arrest. Both synthetic and natural iron chelators have been shown to induce cell cycle arrest, contributing to their anticancer effects on CRC [123, 124]. These chelators primarily affect the cell cycle during the mid‐G1 (Gap 1) phase and the transition from the late G1 to early S (synthesis) phase. During the G1 phase, cells continue to grow and prepare for DNA replication, which occurs in the subsequent S phase [123]. Research indicates that iron is crucial for the transition from the G1 to the S phase, highlighting its role in cell cycle progression [123, 124].

Ribonuclease reductase (RR), an essential enzyme for synthesizing deoxyribonucleotides—the precursors of DNA is—dependent on iron as a cofactor for its enzymatic activity [125]. Therefore, the RR enzymatic activity is highly dependent on the labile iron pool of the cell [125]. Iron chelators such as DFO and Dp44mT can disrupt RR activity by sequestering the labile iron pool, thereby inhibiting the enzyme's function [62, 72, 126]. DFO removes the tyrosyl radical necessary for RR activity, while Dp44mT disrupts RR through interactions with the thiol antioxidant system that maintains RR's structural integrity [48, 59]. Notably, certain natural iron chelators, including extracts from green tea, also exert cell cycle arrest during the G1 phase, leading to apoptosis [79].

### 3.4 Mechanism Related to the Promoting Effects of Iron Chelators on CRC

Interestingly, two studies have reported that iron chelators can stimulate the growth of CRC, revealing specific mechanisms that underlie this paradoxical promoting effect.

#### 3.4.1 Inhibition of Tumor Necrosis Factor (TNF)‐related TRAIL‐Mediated Apoptosis

**FIGURE 4:**
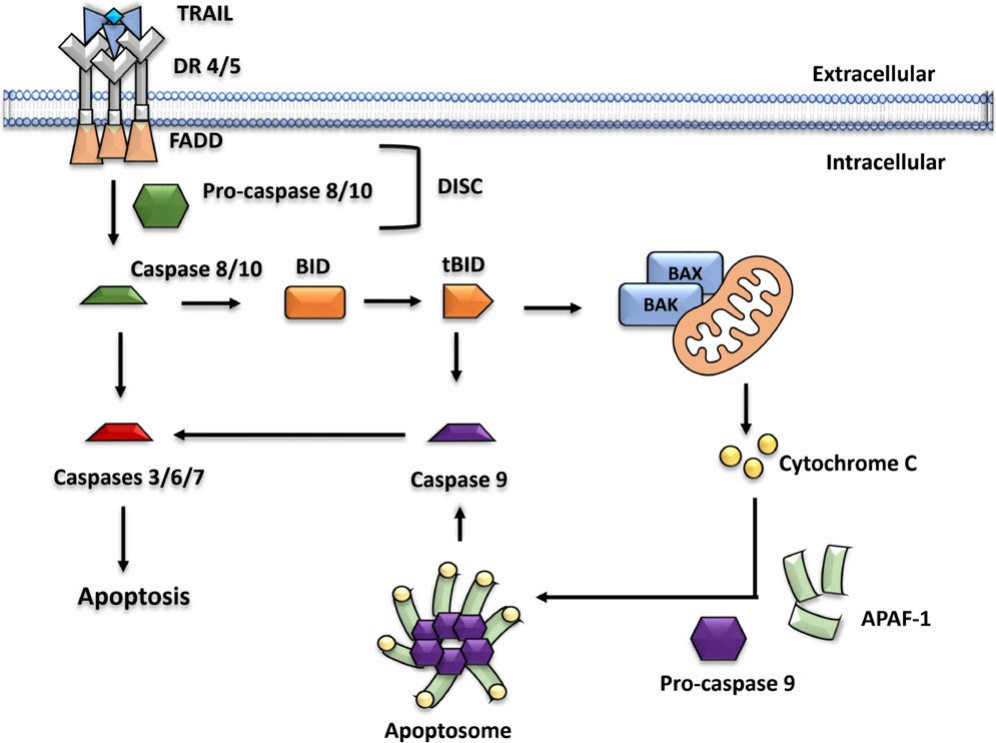
TRAIL‐mediated apoptosis pathway. Adapted from Ref. [131] with permission under a Creative Commons Attribution Licence (CC‐BY) copyright the authors [2023].

TRAIL is a cytokine of the TNF superfamily which is a potential anticancer therapy due to its ability to induce apoptosis in tumor cells without causing any damage to the normal, healthy cells [127]. TRAIL operates through the extrinsic apoptotic pathway, which involves receptor‐mediated interactions. The death receptors DR4 and DR5 bind to TRAIL, initiating the apoptotic cascade [128]. Upon TRAIL binding, these death receptors trimerize, activating the apoptotic signaling pathway by recruiting the adaptor protein Fas‐associated protein with death domain (FADD) along with procaspase 8 or procaspase 10, forming the death‐inducing signaling complex (DISC) [129, 130]. This leads to the activation of caspase 8, which can directly activate effector caspases 3, 6, and 7, triggering either direct apoptosis or indirect caspase 9‐mediated mitochondrial apoptosis pathway (Figure 4).

Interestingly, TRAIL can induce apoptosis by binding to its two death receptors DR4 and DR5, specifically in cancer cells while sparing normal cells. This selective mechanism makes the TRAIL‐mediated apoptotic pathway a promising target for cancer therapy [131, 132]. In this context, DFO has demonstrated proliferative and protective effects on CRC cells by decreasing the caspase‐3 cleavage induced by TRAIL [21]. This caspase‐3 is a critical regulator of programmed cell death, and its inhibition is significant for cell survival [133]. Moreover, DFO activates Akt, which is a key signaling molecule linked with oncogenic receptors necessary to maintain the survival of cancer cells [21]. These combined actions of DFO contribute to increased CRC proliferation and progression. Consequently, it can be concluded that DFO inhibits TRAIL‐mediated apoptosis in CRC, thereby functioning as an antagonist to effective cancer therapies that target this apoptotic pathway.

#### 3.4.2 Activation of Hypoxia Inducible Factors (HIF)

Hypoxia is a critical event in the CRC microenvironment as it activates hypoxia‐inducible factors (HIFs) [134]. HIF is a heterodimer composed of oxygen‐sensitive α subunits (HIF‐ 1α, HIF‐ 2α, HIF‐ 3α) and a β subunit [135]. In CRC, HIF‐1α serves as a mediator of the cellular oxygen pathway, and its overexpression in response to hypoxic conditions results in translocation to the nucleus, where it regulates the expression of target genes [136]. Also, HIF‐1α activates the transcription of several genes involved in CRC pathogenesis, especially in angiogenesis, treatment resistance, and metastasis, thereby enabling cells to adapt to hypoxic conditions [136, 137]. Furthermore, iron influx is elevated while iron efflux is diminished in colon cancer cells, a process that involves various iron metabolism‐related molecules, such as DMT1, TfR1, and hypoxia responsive elements (HRE) [138].

Iron chelators can mimic the hypoxic induction of HIF‐1α, activating transcription by binding to HRE. This activation can initiate the transcription of some genes related to intracellular iron metabolism and angiogenesis, such as TfR and vascular endothelial growth factor (VEGF) [139, 140]. VEGFs are known to enhance cancer metastasis by promoting tumor angiogenesis, stimulating tumor inflammation, and facilitating interactions between cancer cells and vascular endothelium [139, 140]. Hence, activation of HIF‐1α through iron chelation may have tumor‐promoting effects that lead to increased metastasis. An in vitro study with the iron chelator 2,2/−dipyridyl has found that iron chelation can enhance adhesion, invasion, migration, and secretion of metalloproteinases (MMPS) (MMPs are involved in cancer metastases) [73]. This suggests that iron chelation can mimic hypoxic conditions, activating hypoxia‐inducible factors (HIFs) that lead to the transcription of genes related to iron metabolism and angiogenesis, such as transferrin receptor and vascular endothelial growth factor (VEGF) [139, 140]. Consequently, the activation of HIF‐1α by iron chelation may have tumor‐promoting effects, contributing to metastasis in CRC.

## 4 Conclusions and Future Direction

Iron chelation has emerged as a promising therapeutic strategy in various studies related to CRC. The challenges of higher recurrence rates and resistance to chemotherapy highlight the urgent need for novel therapeutic approaches. Iron chelation represents a potential strategy for CRC treatment, although the precise mechanisms of iron chelation in CRC remain inadequately understood.

Diverse colon cancer cell lines across different stages have been explored for the anticancer effects of iron chelation. Deferoxamine is the most commonly used iron chelator in many studies. However, deferasirox, thiosemicarbazone‐based chelators (i.e., Dp44mT), quilamine‐based chelators, and several other novel iron chelators have also been investigated. Notably, various natural plant extracts with iron‐chelating properties have been experimented with as potential treatments for CRC. While the majority of studies indicate that iron chelation can induce antiproliferative effects in CRC cells, some findings suggest the potential for cancer‐promoting effects as well.

Iron chelation can interfere with many hallmarks of CRC, although some cellular targets are yet to be discovered. Mechanisms such as alterations in histone methylation, upregulation of NDRG1, changes in the Wnt/β‐catenin signaling pathway, and modulation of the p53 pathway have been linked to the anticancer effect of iron chelators. However, most studies do not conclusively report the mechanisms underlying these antiproliferative effects, emphasizing the need for further research in this area.

In conclusion, future investigations into iron chelation therapy for CRC should be informed by extensive biological studies, alongside well‐structured clinical trials focusing on the underlying mechanisms of action. When designing clinical trials, the side effects of iron chelation therapy should also be considered, as nausea, vomiting, dizziness, diarrhoea, rash, and muscle cramps are the most typical side effects. It is essential to explore the dual roles of iron chelators, as they can both inhibit tumor growth and promote cancer progression in certain contexts. A comprehensive understanding of how iron chelation impacts key cellular processes, such as histone methylation, cell cycle regulation, and signaling pathways like Wnt/β‐catenin and p53, will refine treatment protocols. Additionally, integrating novel iron chelators, including thiosemicarbazone derivatives and natural plant extracts, may offer unique mechanisms of action that could complement existing therapies. By investigating the interplay between iron metabolism and the tumor microenvironment, researchers can develop targeted approaches that enhance the efficacy of iron chelation while mitigating the risks of promoting cancer progression. These insights will facilitate informed clinical decision‐making and pave the way for robust clinical trials, ultimately optimizing treatment efficacy and potentially improving patient survival rates in those affected by CRC.

## Author Contributions

**Gihani Vidanapathirana:** conceptualization, methodology, software, data curation, investigation, formal analysis, validation, writing – original draft, writing – review and editing. **Md Sajedul Islam:** methodology, software, data curation, formal analysis, validation, writing – review and editing, investigation. **Sujani Gamage:** supervision, writing – review and editing. **Alfred K. Lam:** writing – review and editing, supervision. **Vinod Gopalan:** conceptualization, writing – review and editing, supervision, project administration, validation.

## Disclosure

*Permission to reproduce material from other sources:* Permission to reproduce the images was obtained from the respective journals and/or authors. Documentation of these permissions is provided in the Supporting Information.

## Conflicts of Interest

The authors declare no conflicts of interest.

## Supporting information


File S1.

File S2.

## Data Availability

Data sharing is not applicable to this article as no new data were created or analyzed in this study.
