# Supplementary material for: The Role of Iron Chelation Therapy in Colorectal Cancer: A Systematic Review on Its Mechanisms and Therapeutic Potential
Source: Cancer Med. 2025 Jul 3;14(13):e71019. doi: 10.1002/cam4.71019 (PMC12224056; doi:10.1002/cam4.71019)
Supplement: Supplementary file 1 — File S1. [file CAM4-14-e71019-s001.docx]

Article Retrieval- Iron chelation and colorectal cancers

| Database | Search terms | Mesh terms | No.of Articles |
| --- | --- | --- | --- |
| Embase | (iron AND chelat OR (iron AND chelation) OR (iron AND chelation AND therapy) OR (iron AND chelation AND treatment) OR (excess AND iron AND removal)) AND (colorectal AND cancer OR (colorectal AND neoplasm) OR (colon AND cancer) OR (bowel AND cancer)) | ('iron chelat' OR (('iron'/exp OR iron) AND chelat) OR 'iron chelation'/exp OR 'iron chelation' OR (('iron'/exp OR iron) AND ('chelation'/exp OR chelation)) OR 'iron chelation therapy'/exp OR 'iron chelation therapy' OR (('iron'/exp OR iron) AND ('chelation'/exp OR chelation) AND ('therapy'/exp OR therapy)) OR 'iron chelation treatment' OR (('iron'/exp OR iron) AND ('chelation'/exp OR chelation) AND ('treatment'/exp OR treatment)) OR 'excess iron removal' OR (excess AND ('iron'/exp OR iron) AND removal)) AND ('colorectal cancer'/exp OR 'colorectal cancer' OR (colorectal AND ('cancer'/exp OR cancer)) OR 'colorectal neoplasm'/exp OR 'colorectal neoplasm' OR (colorectal AND ('neoplasm'/exp OR neoplasm)) OR 'colon cancer'/exp OR 'colon cancer' OR (('colon'/exp OR colon) AND ('cancer'/exp OR cancer)) OR 'bowel cancer'/exp OR 'bowel cancer' OR (('bowel'/exp OR bowel) AND ('cancer'/exp OR cancer))) | 160 |
| PubMed | (((((iron chelat') OR (iron chelation)) OR (iron chelation therapy)) OR (iron chelation treatment)) OR (excess iron removal)) AND (((((colorectal cancer') OR (colorectal neoplasm')) OR (colon cancer')) ) OR (bowel cancer')) | (((((iron chelat') OR (iron chelation)) OR (iron chelation therapy)) OR (iron chelation treatment)) OR (excess iron removal)) AND (((((colorectal cancer') OR (colorectal neoplasm')) OR (colon cancer')) ) OR (bowel cancer')) Sort by: Most Recent  ((("iron"[MeSH Terms] OR "iron"[All Fields]) AND "chelat"[All Fields]) OR (("iron"[MeSH Terms] OR "iron"[All Fields]) AND ("chelatable"[All Fields] OR "chelate"[All Fields] OR "chelated"[All Fields] OR "chelater"[All Fields] OR "chelating agents"[Pharmacological Action] OR "chelating agents"[MeSH Terms] OR ("chelating"[All Fields] AND "agents"[All Fields]) OR "chelating agents"[All Fields] OR "chelates"[All Fields] OR "chelator"[All Fields] OR "chelators"[All Fields] OR "chelating"[All Fields] OR "chelation"[All Fields] OR "chelations"[All Fields])) OR (("iron"[MeSH Terms] OR "iron"[All Fields]) AND ("chelation therapy"[MeSH Terms] OR ("chelation"[All Fields] AND "therapy"[All Fields]) OR "chelation therapy"[All Fields])) OR (("iron"[MeSH Terms] OR "iron"[All Fields]) AND ("chelatable"[All Fields] OR "chelate"[All Fields] OR "chelated"[All Fields] OR "chelater"[All Fields] OR "chelating agents"[Pharmacological Action] OR "chelating agents"[MeSH Terms] OR ("chelating"[All Fields] AND "agents"[All Fields]) OR "chelating agents"[All Fields] OR "chelates"[All Fields] OR "chelator"[All Fields] OR "chelators"[All Fields] OR "chelating"[All Fields] OR "chelation"[All Fields] OR "chelations"[All Fields]) AND ("therapeutics"[MeSH Terms] OR "therapeutics"[All Fields] OR "treatments"[All Fields] OR "therapy"[MeSH Subheading] OR "therapy"[All Fields] OR "treatment"[All Fields] OR "treatment s"[All Fields])) OR (("iron overload"[MeSH Terms] OR ("iron"[All Fields] AND "overload"[All Fields]) OR "iron overload"[All Fields] OR ("excess"[All Fields] AND "iron"[All Fields]) OR "excess iron"[All Fields]) AND ("removability"[All Fields] OR "removal"[All Fields] OR "removals"[All Fields] OR "remove"[All Fields] OR "removed"[All Fields] OR "removement"[All Fields] OR "remover"[All Fields] OR "removers"[All Fields] OR "removes"[All Fields] OR "removing"[All Fields]))) AND ("colorectal neoplasms"[MeSH Terms] OR ("colorectal"[All Fields] AND "neoplasms"[All Fields]) OR "colorectal neoplasms"[All Fields] OR ("colorectal"[All Fields] AND "cancer"[All Fields]) OR "colorectal cancer"[All Fields] OR ("colorectal neoplasms"[MeSH Terms] OR ("colorectal"[All Fields] AND "neoplasms"[All Fields]) OR "colorectal neoplasms"[All Fields] OR ("colorectal"[All Fields] AND "neoplasm"[All Fields]) OR "colorectal neoplasm"[All Fields]) OR ("colonic neoplasms"[MeSH Terms] OR ("colonic"[All Fields] AND "neoplasms"[All Fields]) OR "colonic neoplasms"[All Fields] OR ("colon"[All Fields] AND "cancer"[All Fields]) OR "colon cancer"[All Fields]) OR ("intestinal neoplasms"[MeSH Terms] OR ("intestinal"[All Fields] AND "neoplasms"[All Fields]) OR "intestinal neoplasms"[All Fields] OR ("bowel"[All Fields] AND "cancer"[All Fields]) OR "bowel cancer"[All Fields])) | 152 |
| Medline (Via Web of Science) | ( iron chelation or iron chelation therapy or iron chelation treatment or excess iron removal ) AND ( colorectal cancer or colon cancer or bowel cancer or rectal cancer ) | ( iron chelation or iron chelation therapy or iron chelation treatment or excess iron removal ) AND ( colorectal cancer or colon cancer or bowel cancer or rectal cancer ) | 25 |
| Scopus |  | ( TITLE-ABS-KEY ( iron AND chelation ) OR TITLE-ABS-KEY ( iron AND chelation AND therapy ) OR TITLE-ABS-KEY ( iron AND chelation AND treatment ) OR TITLE-ABS-KEY ( excess AND iron AND removal ) AND TITLE-ABS-KEY ( colorectal AND cancers ) OR TITLE-ABS-KEY ( colon AND cancer ) OR TITLE-ABS-KEY ( colorectal AND neoplasm ) OR TITLE-ABS-KEY ( bowel AND cancer ) ) | 93 |
